# Ischemic Stroke as the Presenting Feature of Anterior STEMI Complicated by LV Thrombus and Pseudoaneurysm

**DOI:** 10.1016/j.jaccas.2026.109929

**Published:** 2026-08-20

**Authors:** Ibrahim Hasan, Nebras Hasan, Pierre Sfeir, Noura El Sayegh, Marwan M. Refaat, Ziyad Ghazzal

**Affiliations:** a Division of Cardiology, Department of Internal Medicine, American University of Beirut Medical Center, Beirut, Lebanon; b Division of Cardiothoracic Surgery, Department of Surgery, American University of Beirut Medical Center, Beirut, Lebanon; c Division of Anatomic Pathology, Department of Pathology and Laboratory Medicine, American University of Beirut Medical Center, Beirut, Lebanon

**Keywords:** delayed-presentation STEMI, ischemic stroke, left ventricular pseudoaneurysm, left ventricular thrombus, mechanical complications

## Abstract

**Background:**

Delayed presentation of anterior ST-segment elevation myocardial infarction (STEMI) increases the risk of thromboembolic and mechanical complications.

**Case Summary:**

We report a 41-year-old man who presented with transient right upper extremity weakness and dysarthria and was found to have electrocardiographic evidence of an old anterior myocardial infarction. He experienced chest pain 3 days earlier without seeking medical attention. Cardiac catheterization revealed thrombotic lesions involving the left anterior descending artery and diagonal bifurcation. Echocardiography demonstrated moderately reduced left ventricular systolic function with a large apical thrombus. Brain magnetic resonance imaging showed a small acute ischemic infarct. He was managed medically with antithrombotics and heart failure therapy. Eight weeks later, regular follow-up imaging revealed severe left ventricular dysfunction, aneurysmal remodeling, and pseudoaneurysm formation, requiring urgent surgical repair.

**Discussion:**

This case highlights the dynamic progression from thromboembolic to mechanical complications following delayed-presentation STEMI and underscores the importance of early recognition, multimodality imaging, and close follow-up.

**Take-Home Messages:**

Delayed-presentation anterior STEMI may first manifest as acute ischemic stroke because of left ventricular thrombus. Close follow-up and multimodality imaging are essential to detect late mechanical complications such as left ventricular pseudoaneurysm.

## Visual Summary

**Figure:**
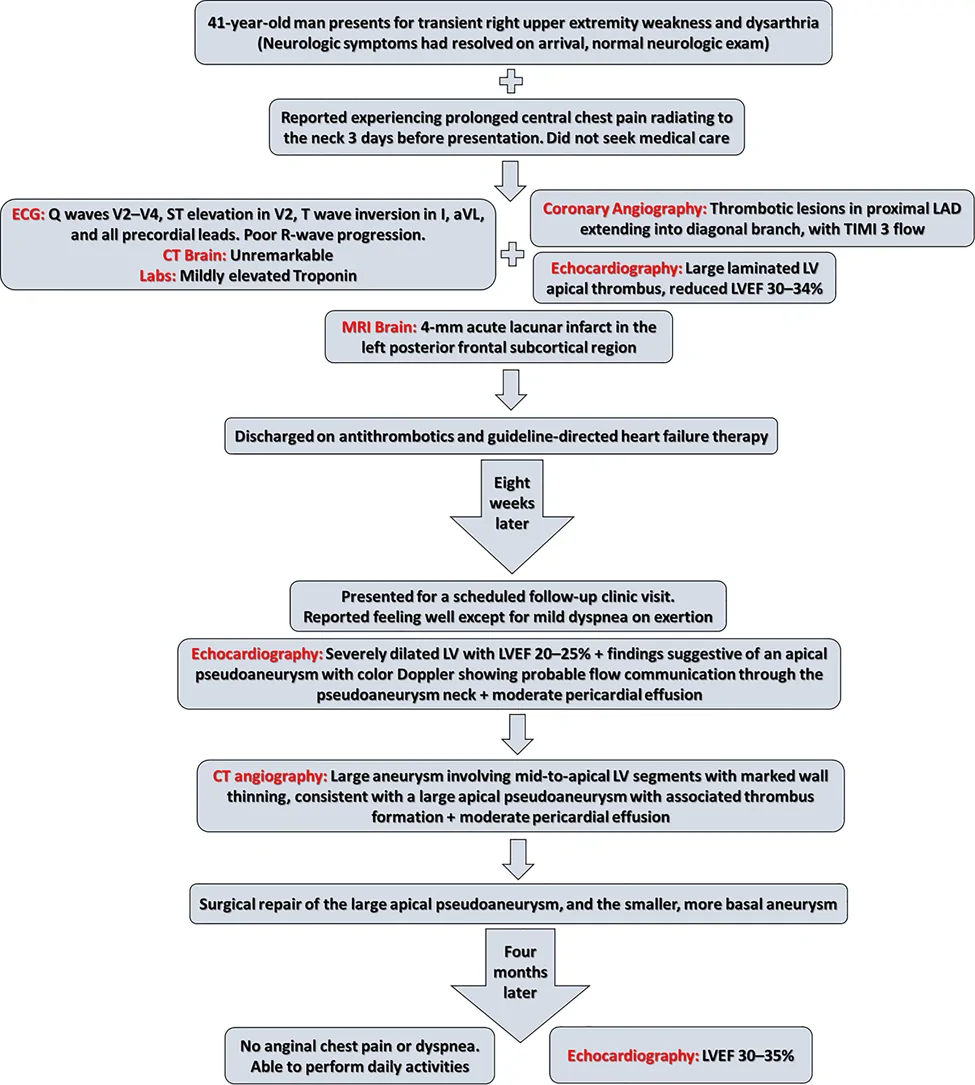

Acute ischemic stroke is an uncommon but clinically important complication of myocardial infarction, often related to left ventricular thrombus formation following large anterior infarctions.^1^ In ST-segment elevation myocardial infarction (STEMI), delayed presentation and delayed reperfusion increase the risk of thromboembolic and mechanical complications, including left ventricular thrombus, systemic embolization, and ventricular aneurysm formation.^1^^,^^2^ These aneurysmal segments provide a substrate for thrombus formation and subsequent embolic events. In rare cases, progression to myocardial rupture may result in the formation of a left ventricular pseudoaneurysm, a life-threatening condition with a high risk of fatal rupture if not promptly recognized.^2^^,^^3^

We report a case in which acute ischemic stroke was the initial manifestation of delayed-presentation anterior STEMI complicated by left ventricular thrombus formation, followed by aneurysmal remodeling and pseudoaneurysm formation.

## Case Presentation

### History of presentation

A 41-year-old man presented to the emergency department with an acute transient neurological episode. He reported a sudden weakness of the right upper extremity, accompanied by a sensation of impending loss of consciousness. This episode was associated with transient dysarthria. By the time he presented to the emergency department, his neurological examination was unremarkable. Three days before presentation, the patient experienced an episode of prolonged chest pain, described as central in location and radiating to the neck that lasted several hours; however, he did not seek medical attention at that time.

### Past medical history

His medical history was notable for smoking and prior resection of a right thigh angioma.

### Differential diagnosis

The differential diagnosis included a transient ischemic attack or acute stroke. Given the recent episode of prolonged chest pain, a recent acute coronary syndrome was also considered.

### Investigations

Electrocardiography (ECG) demonstrated Q waves in leads V_2_-V_4_, with 1.5 mm ST-segment elevation in V_2_ and T-wave inversion across all precordial leads, as well as in leads I and aVL, with poor R-wave progression (Figure 1), suggestive of an old anterior myocardial infarction. Initial high-sensitivity troponin was mildly elevated at 0.037 ng/mL (reference range: up to 0.030 ng/mL). A noncontrast computed tomography (CT) scan of the brain was unremarkable.

**Figure 1:**
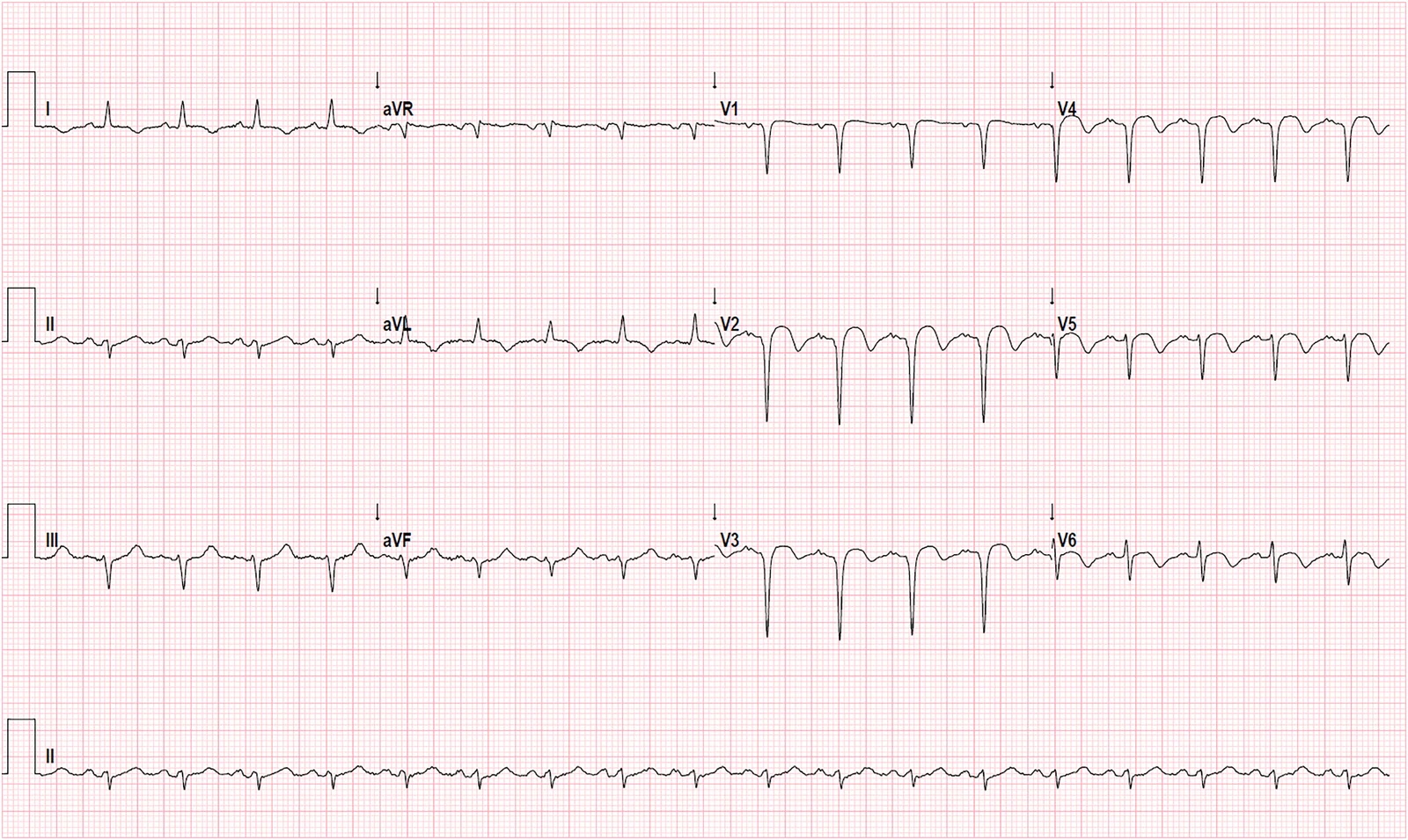
Electrocardiogram Demonstrating Q Waves in Anterior Leads With 1.5 mm ST-Segment Elevation in V_2_ and T-Wave Inversion Across All Precordial Leads, Leads I and aVL, With Poor R-Wave Progression

### Management

The patient was loaded with aspirin and clopidogrel and initiated on an intravenous heparin infusion. Coronary angiography showed thrombotic lesions in the proximal left anterior descending artery extending into the diagonal branch with TIMI flow grade 3 (Figure 2). Given the history of prolonged chest pain 3 days earlier and ECG evidence of an established anterior infarction with Q waves, the angiographic pattern was most consistent with spontaneous reperfusion following an initially occlusive thrombotic event, most likely because of plaque rupture or plaque erosion. Although intravascular imaging could have provided further insight into the underlying mechanism by distinguishing plaque rupture from plaque erosion, it was not performed because the findings were unlikely to alter immediate management in the setting of preserved epicardial flow and an established infarction.

**Figure 2:**
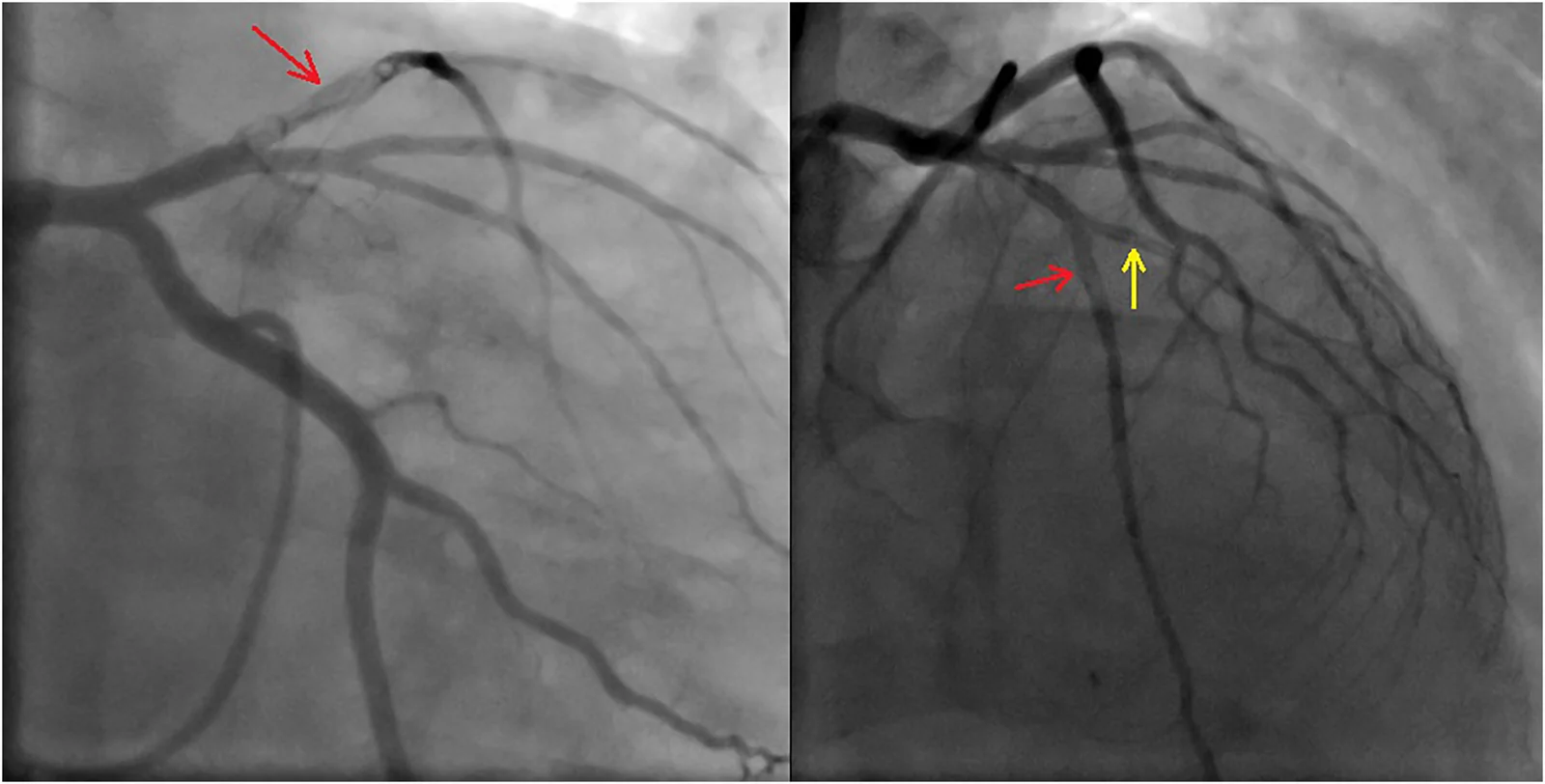
Coronary Angiography Showing Filling Defects Consistent With Thrombotic Lesions in the Proximal Left Anterior Descending Artery (Red Arrow) and Diagonal Branch (Yellow Arrow), With Preserved TIMI Flow Grade 3

Transthoracic echocardiography demonstrated moderately reduced left ventricular systolic function, with an estimated ejection fraction of 30%-34%. There were extensive regional wall motion abnormalities characterized by akinesis of the basal anterior, basal anterior septal, mid anterior, mid anterior septal, mid inferior septal, mid inferior, apical anterior, apical septal, apical inferior segments, and the apex. A large laminated apical left ventricular thrombus was noted, measuring approximately 1.30 × 2.30 cm (Figure 3). Therapeutic enoxaparin was initiated. Brain magnetic resonance imaging showed a small, 4-mm acute lacunar infarct in the left posterior frontal subcortical region. Magnetic resonance angiography of the brain and neck demonstrated no vascular abnormalities. The patient was discharged on antithrombotics and guideline-directed heart failure therapy.

**Figure 3:**
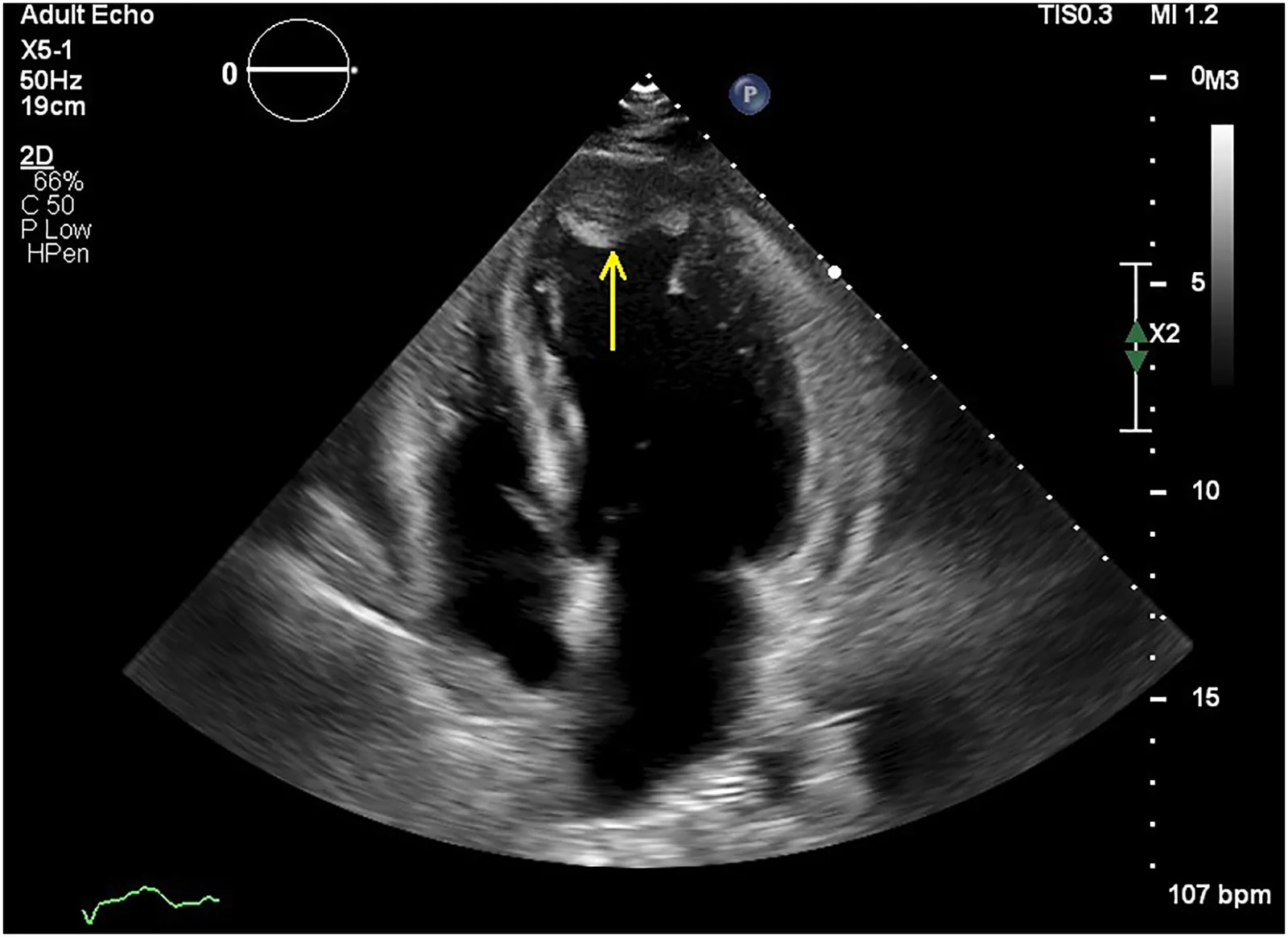
Transthoracic Echocardiography Demonstrating a Large Apical Left Ventricular Thrombus (Yellow Arrow)

### Outcome and follow-up

The patient presented for a scheduled follow-up clinic visit 8 weeks later. He reported feeling well except for mild dyspnea on exertion. Unexpectedly, echocardiography showed severely impaired left ventricular systolic function, with an estimated ejection fraction of 20%-25%. There was aneurysmal dilatation involving the mid anterolateral, apical anterior, apical lateral segments, and the apex (Figure 4). The study was also suggestive of a left ventricular pseudoaneurysm, with color Doppler demonstrating probable flow communication through its neck (Figure 5), as well as a moderate pericardial effusion without evidence of cardiac tamponade.

**Figure 4:**
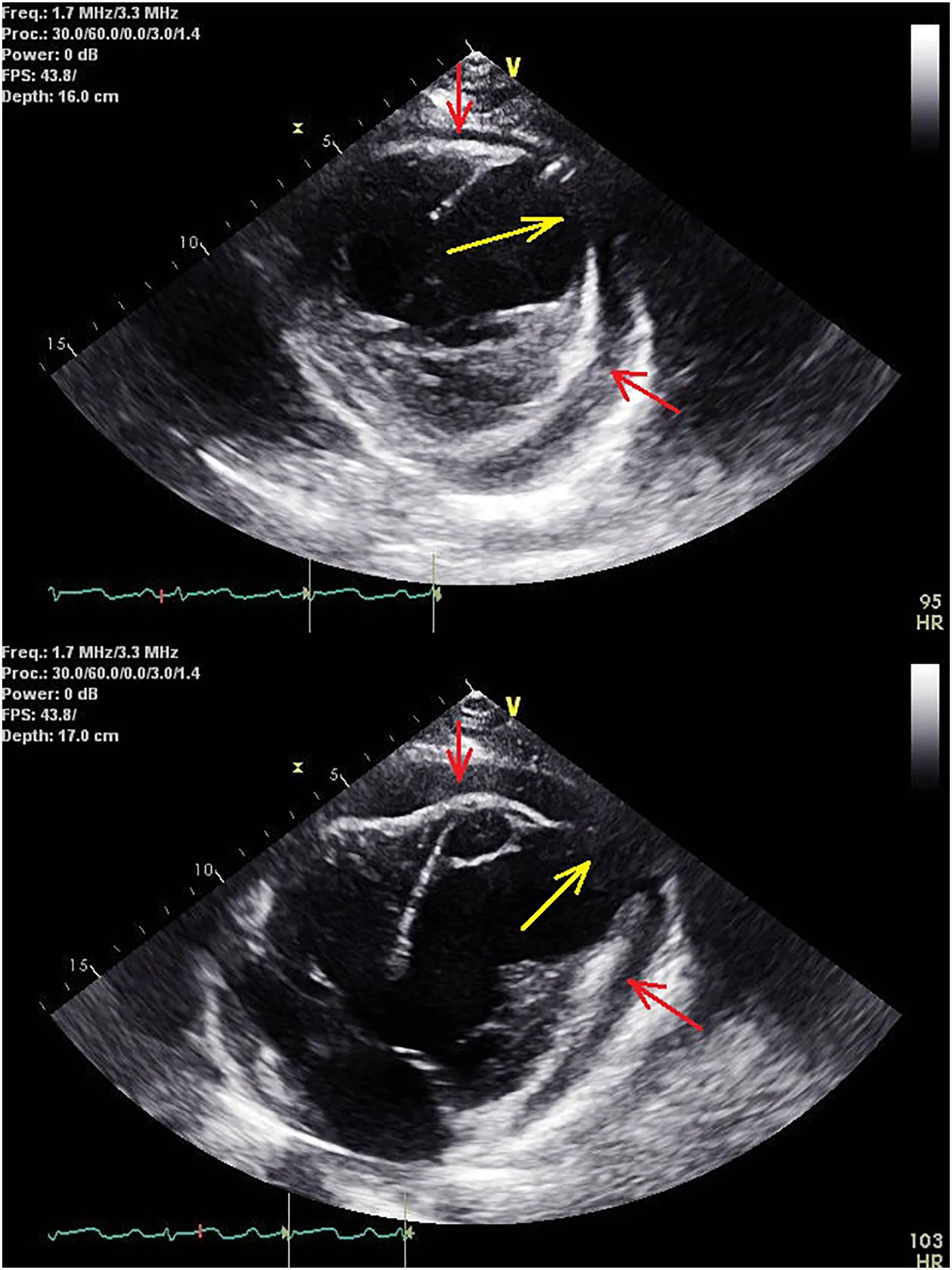
Follow-Up Transthoracic Echocardiography Showing Aneurysmal Remodeling of the Anterior and Apical Segments (Yellow Arrow) and Pericardial Effusion (Red Arrow)

**Figure 5:**
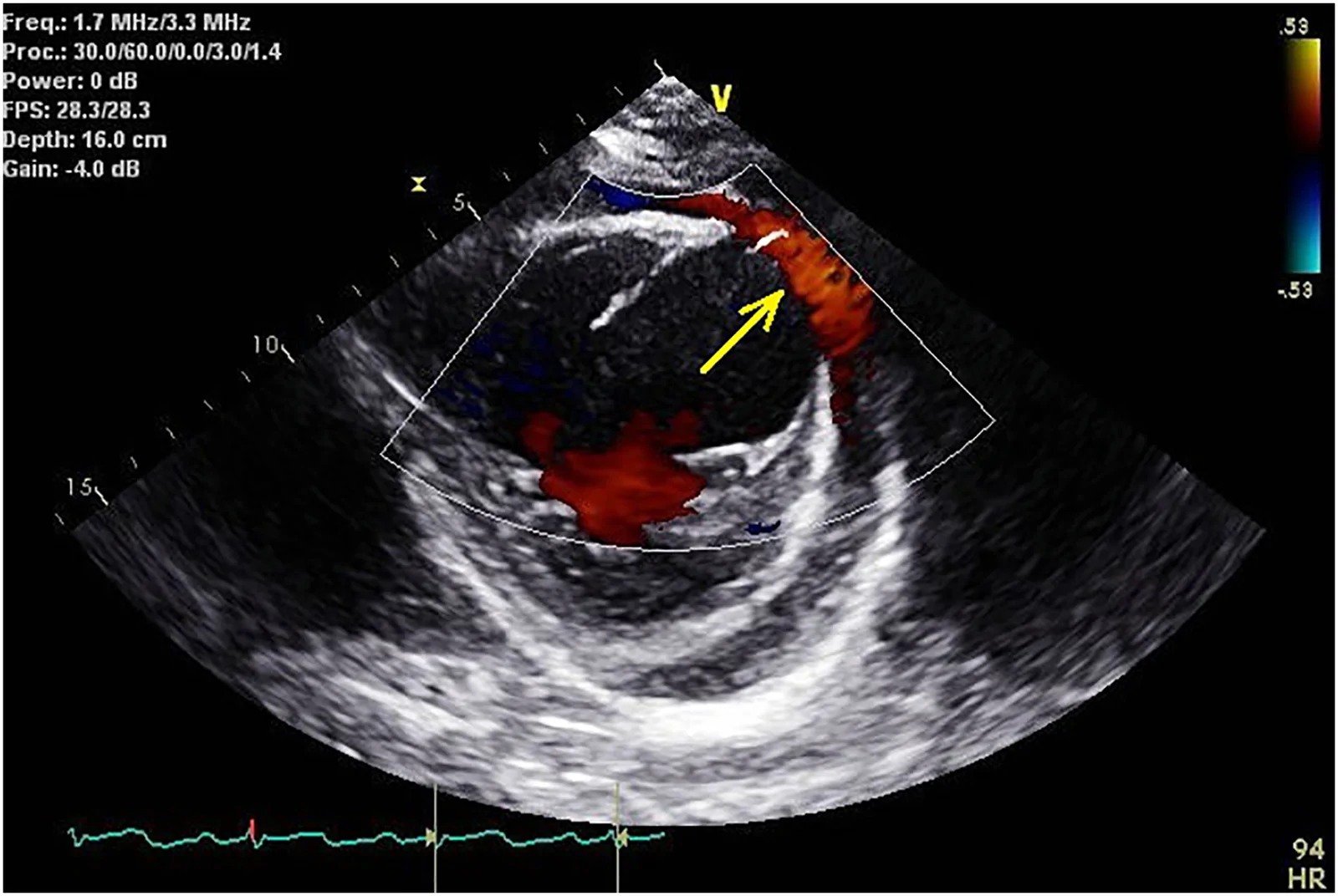
Color Doppler Echocardiography Demonstrating Left Ventricular Pseudoaneurysm With Apparent Flow Communication Through the Pseudoaneurysm Neck (Yellow Arrow)

CT angiography of the chest demonstrated a large aneurysm involving the mid-to-apical left ventricular segments with marked wall thinning, consistent with a large pseudoaneurysm involving the left ventricular apex with associated thrombus formation, along with a moderate pericardial effusion (Figure 6). The patient underwent surgical repair of a large apical aneurysm, which was suspicious for a pseudoaneurysm, and a smaller, more basal aneurysm (Figure 7). Pathologic examination confirmed the diagnosis of an apical pseudoaneurysm, characterized by the absence of myocardial tissue in the wall (Figure 8).

**Figure 6:**
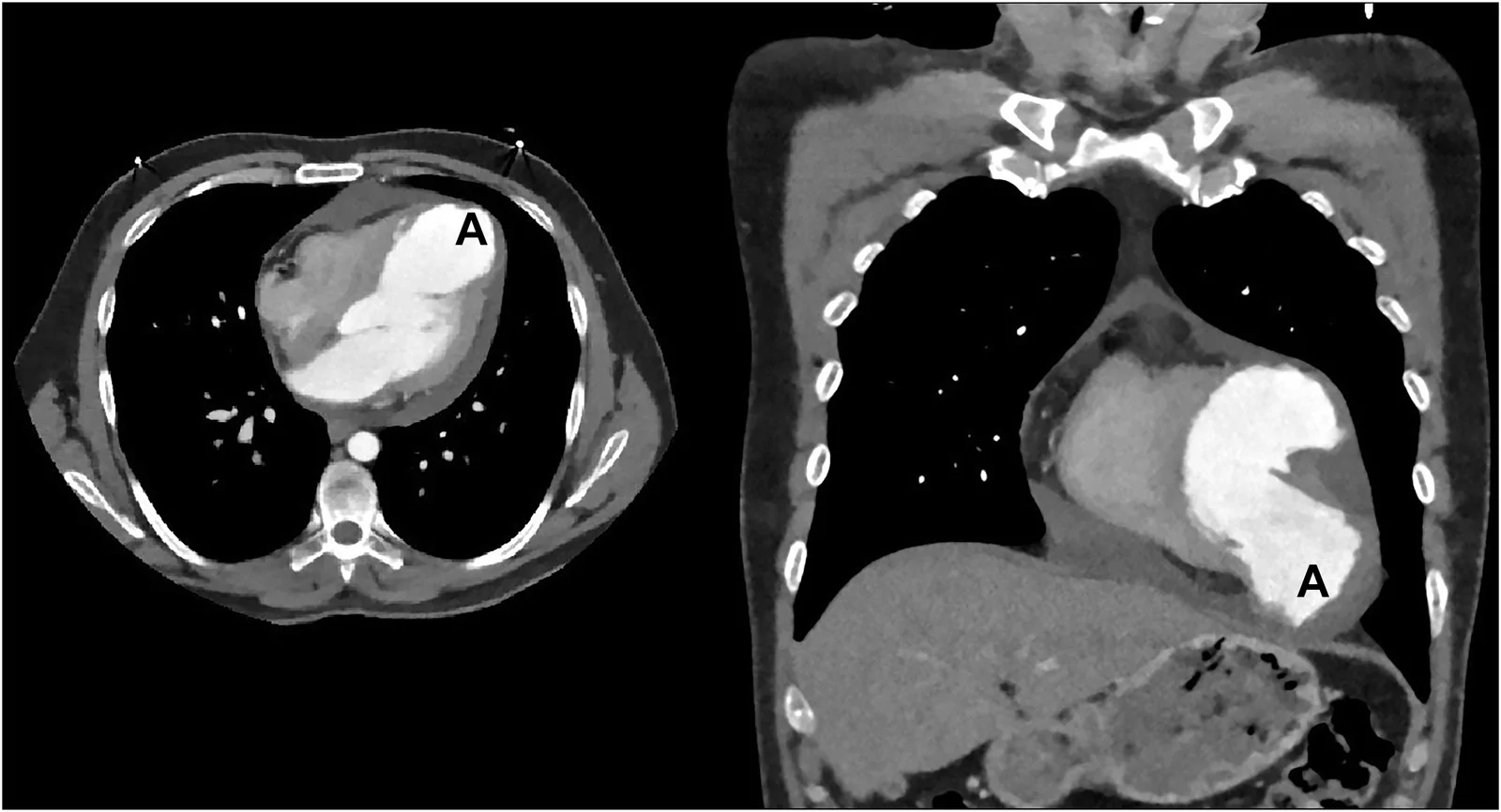
Computed Tomography Angiography Showing a Large Aneurysm (A) Involving the Mid-to-Apical Left Ventricular Segments With Marked Wall Thinning, Consistent With a Large Pseudoaneurysm Involving the Left Ventricular Apex, Along With a Moderate Pericardial Effusion

**Figure 7:**
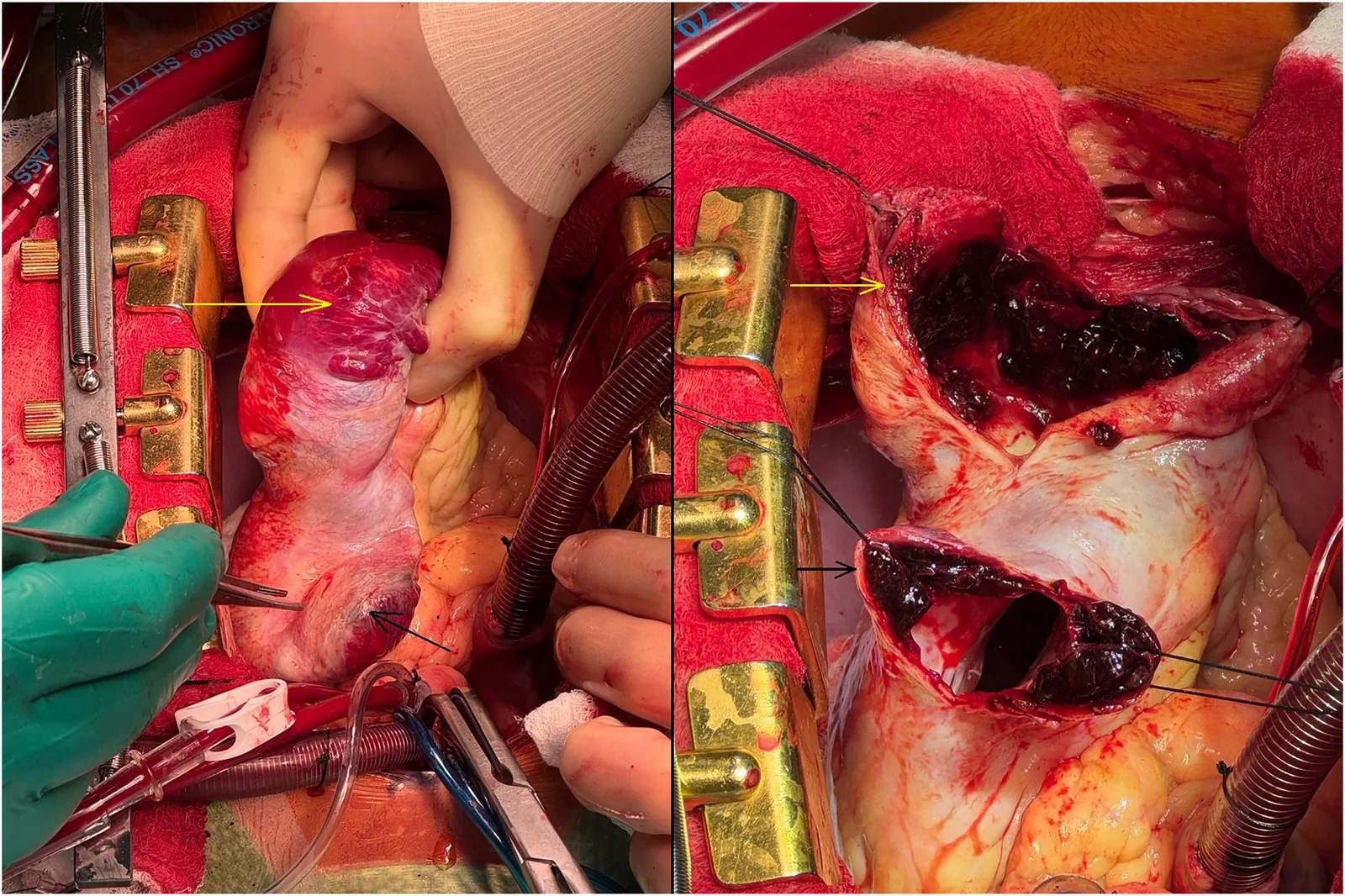
Intraoperative Visualization of a Large Apical Pseudoaneurysm (Yellow Arrow) and a Smaller Basal Left Ventricular Aneurysm (Black Arrow), Both Containing Thrombus

**Figure 8:**
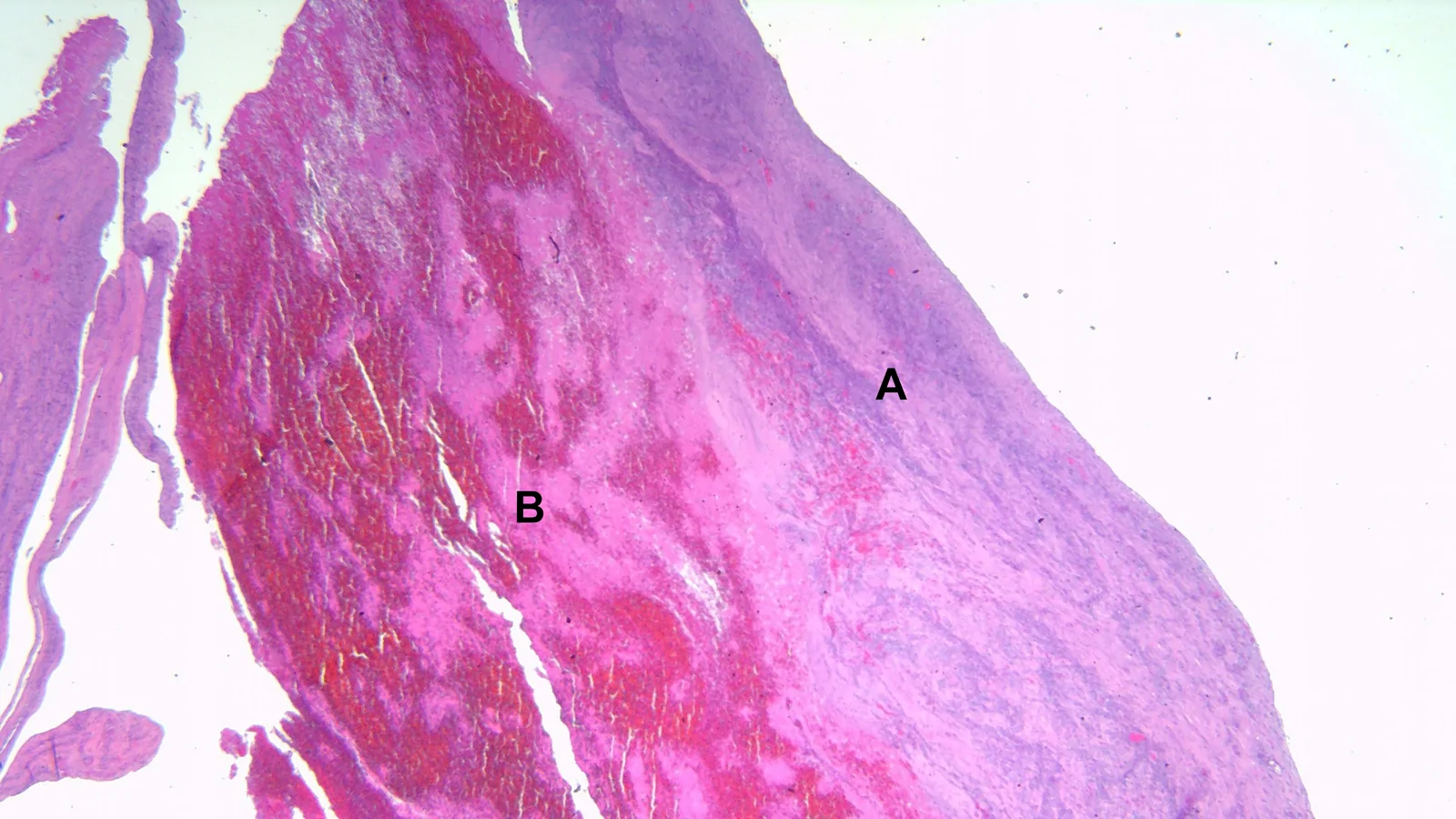
Histopathologic Examination of the Resected Apical Segment (A) Myxoid degeneration and fibrosis, (B) organizing thrombus (H&E ×100).

Approximately 4 months after aneurysm repair, echocardiography demonstrated an estimated ejection fraction of 30%-35%. He reported no anginal chest pain or dyspnea and was able to perform his daily activities without limitation.

## Discussion

Mechanical complications of STEMI represent high-acuity, time-sensitive conditions associated with substantial morbidity and mortality.^2^ Although the widespread adoption of early reperfusion therapy has significantly reduced their incidence, patients with large infarctions or delayed presentations remain at considerable risk for both thromboembolic and mechanical sequelae.^2^ Moreover, even when timely epicardial reperfusion is achieved, severe microvascular injury and impaired tissue-level reperfusion may sustain large infarct size and predispose to thromboembolic and mechanical complications. A complex clinical scenario may arise when these complications coexist, particularly during the subacute phase of an unrecognized infarction in which the underlying pathology has already evolved. This case illustrates the diagnosis of a cardioembolic neurologic event following a delayed presentation of a large anterior myocardial infarction, followed by progression to a potentially fatal contained cardiac rupture, highlighting the dynamic interplay between thromboembolic and mechanical complications following myocardial infarction.

Systemic embolism presenting as acute ischemic stroke may be the initial manifestation of a silent or unrecognized myocardial infarction.^4^^,^^5^ Left ventricular thrombus typically develops within days to weeks after acute injury,^6^ with large anterior infarctions conferring the highest risk because of extensive apical involvement.^1^^,^^2^ Accordingly, the presence of stroke in conjunction with regional wall motion abnormalities should prompt consideration of a recent, previously undiagnosed transmural myocardial infarction.^1^^,^^2^ Notably, the stroke in our patient was a subcortical lacunar infarct; although most lacunar strokes are traditionally attributed to cerebral small-vessel disease rather than an embolic mechanism,^7^ a minority may still arise from embolic mechanisms and therefore warrant clinical correlation.^8^^,^^9^

As myocardial remodeling progresses, the infarcted wall may evolve into a true left ventricular aneurysm.^2^ These aneurysms consist of thinned, fibrotic myocardium that maintains structural continuity with the ventricular wall; they are typically characterized by a wide neck and carry a relatively low risk of rupture.^2^^,^^3^ Nevertheless, the noncontractile aneurysmal segment perpetuates blood stasis and serves as a substrate for persistent thrombus formation,^2^ and it may represent a structural bridge toward subsequent mechanical complications.^10^

In contrast, a left ventricular pseudoaneurysm represents a contained free-wall rupture in which blood is confined by adherent pericardium or fibrous tissue rather than myocardium.^2^^,^^3^ It is classically characterized by a narrow neck and absence of normal myocardial layers, conferring a markedly elevated risk of catastrophic rupture.^3^ Given the high mortality associated with untreated pseudoaneurysms, early recognition using multimodality imaging and prompt surgical intervention are critical. Diagnosis is best established with multimodality imaging: Transthoracic echocardiography is the first-line test, whereas color Doppler helps demonstrate the characteristic flow communication through the pseudoaneurysm neck; cardiac CT is particularly valuable for confirming the diagnosis, defining the neck, cavity, and rupture site, and distinguishing pseudoaneurysm from a true aneurysm.^3^ Cardiac magnetic resonance is also a valuable imaging modality in this setting. The high tissue characterization capability of cardiac magnetic resonance enables precise differentiation between pericardium, thrombus, and myocardial tissue.^3^ Additionally, its excellent spatial resolution allows for detailed assessment of the size and exact location of the rupture.^3^

## Conclusions

This case highlights how an acute ischemic stroke can be the first manifestation of an undiagnosed STEMI, with subsequent progression to a life-threatening pseudoaneurysm. It underscores the need for careful cardiovascular evaluation in patients with embolic stroke and suggestive ECG or echocardiographic findings, particularly when a prior infarction may have gone unrecognized.

**Take-Home Messages**

• Delayed-presentation anterior ST-segment elevation myocardial infarction may first manifest as acute ischemic stroke because of left ventricular thrombus.
• Close follow-up and multimodality imaging are essential to detect late mechanical complications such as left ventricular pseudoaneurysm.

**Visual Summary:**
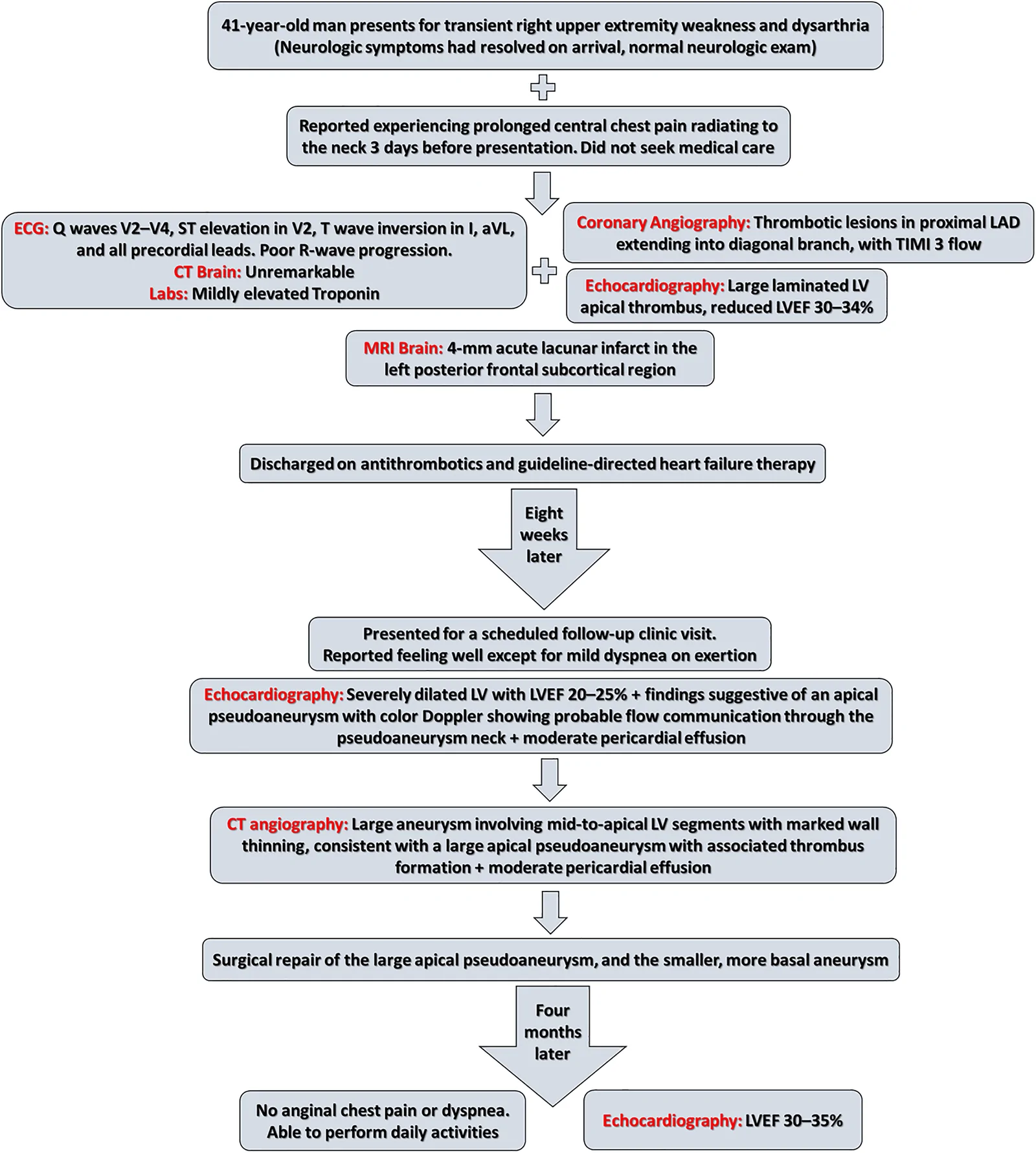
Clinical Timeline of Delayed-Presentation Anterior ST-Segment Elevation Myocardial Infarction Complicated by Left Ventricular Thrombus, Ischemic Stroke, and Left Ventricular Pseudoaneurysm CT = computed tomography; ECG = electrocardiography; LAD = left anterior descending artery; LVEF = left ventricular ejection fraction; MRI = magnetic resonance imaging.

## Funding Support and Author Disclosures

The authors have reported that they have no relationships relevant to the contents of this paper to disclose.
